# Decomposing the change in the cognitive function gap between older men and women over time in China: The Chinese Longitudinal Healthy Longevity Survey

**DOI:** 10.7189/jogh.13.04143

**Published:** 2023-11-22

**Authors:** Na Cao, Xin Zeng, Peigang Wang

**Affiliations:** Department of Social Medicine and Health Management, School of Public Health, Wuhan University, Wuhan, Hubei, China

## Abstract

**Background:**

This study explored how modifiable social determinants of cognitive function can influence these gender gaps.

**Methods:**

We utilized six waves of the Chinese Longitudinal Healthy Longevity Survey (CLHLS), including 56,127 individuals aged 65+. The Kitagawa-Oaxaca-Blinder decomposition approach was used for the analysis.

**Results:**

Older women consistently had lower average levels of cognitive function than men in each period, but the gap is narrowing. From 2002 to 2018, the gender gap in cognitive function decreased by 1.45 (95% confidence interval (CI) = -1.843, -1.097) points. The coefficients for the endowment effects decreased from 0.387 (95% CI = -0.563, -0.211) to 1.789 (95% CI = -2.471, -1.107) from 2005 to 2018. Lifestyle changes, social participation, and physical health factors significantly contributed to explaining the changes in gender gaps in cognitive function.

**Conclusions:**

Among these contributing factors, lifestyle, social participation, and physical health have emerged as pivotal elements in reducing the gender gap in cognitive function. Targeted interventions for these variables are essential among older women to narrow the cognitive gender gaps effectively.

Despite their advantage in mortality rates, women encounter a cognitive disadvantage, commonly termed as the male-female “health-survival” paradox in the domain of cognitive function. Previous studies consistently revealed lower cognitive function levels among women compared to men in China [1-3]. For instance, a cross-sectional study reported a dementia prevalence of 7.04% among women and 4.97% among men in China [4]. However, recent studies have shown that gender gaps in cognitive function, once observed in earlier cohorts, have diminished among older adults [5,6], suggesting a gradual decrease in gender gaps in cognitive function over time [7]. Therefore, it is imperative to investigate the determinants that have led to this shift to comprehend the risk factors thoroughly and devise more efficient interventions. 

The decisive question that arises pertains to the cause of the gaps in cognitive performance between older men and women. Due to the improvement of social economy, a growing rate of the aging population, modifications of family structures in China, and risk factors of cognitive function may also change, which will in turn significantly affect gender gaps in cognitive function over time. Exploring the correlation between the alterations in the social determinants of cognitive function can be instrumental in advancing interventions for cognitive function and reducing the gender gaps among older people. This is achieved by providing avenues for intervention that allow us to manipulate these changes.

Numerous prior studies have observed gender gaps in cognitive function and provided diverse explanations for these gaps. These risk factors include social structures, behaviors, and social-psychological factors [8]. Structures of social inequality include socioeconomic status [9-12], labor force participation [13], occupational type and the nature of related tasks [14,15], and marital status [16,17]. Meanwhile, lifestyle behaviours such as physical activity [18], smoking habits [19], exercise [20], diet [21], and health care services seeking [22] have also been identified as risk factors. Additionally, psychological factors, including impaired mental health (particularly depression), have been associated with cognitive impairment [12,23,24].

Numerous factors interact in a complex manner to progressively narrow the gender gap in cognitive function. For instance, as socio-economic development advances, influential parameters like the augmentation of women's social status, a marked increase in the number of educated women, a significant rise in female employment rates, and the enhancement of women's social rights and benefits all holistically contribute to improving cognitive functions among women [25,26]. However, there exists a limited comprehension of cognitive function gaps between older men and women, and only a few studies have delved into how changes in these risk factors may affect gender gap in cognitive function.

Considering this, employing decomposition techniques can provide a valuable avenue for further exploring this issue. A recent study on older Japanese individuals utilized decomposition analysis to examine gender gaps in cognitive function and decline. The findings revealed that these gaps can be attributed to distinct individual attributes and social determinants, with men appearing to be more engaged in activities that accumulate intellectual experiences through education and occupation [27]. Furthermore, the cognitive function gaps between older men and women may have changed over time due to the influence of modifiable social variables. It could be questioned whether this change is caused by compositional shifts (differential changes in the endowments of the groups) or changes in the coefficient contributions. Due to technological advancements, we can currently utilize a longitudinal decomposition technique to examine the aforementioned concern.

This study aimed investigate gender gaps in cognitive function among older Chinese adults by employing a longitudinal data decomposition method that incorporates social determinants. Furthermore, we aimed to discern the factors influencing changes in the gender gap in cognitive function and explore their underlying causes. To mitigate the cognitive function gaps, we applied the Kitagawa-Oaxaca-Blinder (KOB) decomposition to the longitudinal data. Significantly, our study represents the pioneering utilization of this longitudinal decomposition method to comprehensively analyze and provide empirical evidence on the evolving gender differences in cognitive function among older adults in China.

## METHODS

### Data sources

Data were obtained from the Chinese Longitudinal Healthy Longevity Survey (CLHLS). The original baseline survey was carried out in 1998, and follow-up waves were conducted in 2000, 2002, 2005, 2008-2009, 2011-2012, 2014, and 2017-2018. The survey was carried out via a random selection process spanning half of the counties and cities within 22 provinces in China, effectively covering approximately 85% of the country's population. To ensure a representative sample, a multistage, stratified random cluster sampling approach was employed [28]. The survey gathered extensive data on health status (for example, cognitive function) and its determinants among older adults. The representativeness of the dataset and the wealth of variables render it suitable for our study. The distribution of age and gender within the CLHLS is uniform distribution of age and gender, providing a substantial number of respondents. Initially, the survey exclusively interviewed people who were 80 years of age or older, but starting in 2002, people who were 65 years of age or older were also surveyed. To ensure the representativeness of the data, a specific number of fresh respondents was selected for each survey to complement the existing sample group. It has been questioned if centenarians, those who 106 years of age are and older, provide accurate answers to questionnaires [28]. The CLHLS had six waves between 2002 and 2018, with samples ranging in age from 65 to 105. A total of 56,127 valid observations emerged after samples with crucial data missing for the study's variables were eliminated.

### Variables

#### Dependent variables

Cognitive function in this study was measured by the Mini-Mental State Examination (MMSE), a brief cognitive function assessment developed by Folstein et al. [29]. The following five domains of the MMSE are applied to determine scores:

1. Percentage of correct answers to questions about the year, month, day of the week, and date;
2. Mark attained on a five-times serial subtraction task (counting backwards from 100 by sevens);
3. Immediate recall of a list of ten words;
4. Delayed recall of the same list of ten words;
5. An elementary drawing task.

One point is awarded for each correct response, while no points are awarded for incorrect responses. The scores from all the items are summed, resulting in a range of scores from 0 to 30. Higher scores indicate higher levels of cognitive function.

#### Independent variables

Based on previous research, the independent variables included age, education, occupation before retirement, marital status, current residence, co-residence, disability, social participation (doing housework, participating in outdoor activities, gardening and raising pets, reading books and newspapers, raising domestic animals, playing cards or mahjong, watching TV and listening to the radio, and participating in social activities), social contact (visiting from siblings, visiting from children, talking to someone, having someone to talk to about what's on your mind, having someone to help in trouble, having someone to take care of you when sick), lifestyle (smoking, drinking, exercising), and life satisfaction (Appendix S1 in the **Online Supplementary Document**).

### Statistical analysis

In this study, we employed a counterfactual method called the KOB decomposition to investigate how much the gaps in changes in cognitive function between older men and women can be clarified by gaps in the distributions of the explanatory variables. This KOB decomposition is a widely utilized methodology for examining outcomes across different groups, such as sex and race [30-32]. This approach allows researchers to understand the factors contributing to outcomes between these groups. The KOB technique, recently developed by Kröger and Hartmann [33], fits longitudinal data and enables an analysis of gaps in mean cognitive function based on a mixed-effects regression model. This model incorporates a time dimension and is based on the variances in means of the relevant variables, somewhat like the conventional KOB technique. In other words, we must settle on a period to use as the model’s reference. In this study, we used the year 2002 as a reference. We employed the Interventionist technique for its decomposition [33], which takes on a three-fold form. The variation in cognitive function gaps between elderly men and women is broken down into the variation of three component effects: endowments, coefficients, and interaction terms (Appendix S2 in the **Online Supplementary Document**). The endowments effect signifies the portion of the differences attributable to group gaps in the predictors, which is of primary interest to researchers. The coefficients effect gauges the contribution of gaps in the coefficients. Meanwhile, the interaction effect accounts for the part of the gaps that are due to the interaction of the groups’ different characteristics and coefficients [33].

All statistical analyses were conducted using STATA version 17.0 (StataCorp LLC, College Station, Texas, USA). We employed the bootstrap method to obtain a confidence interval (1000 iterations). This approach allowed us to calculate the decomposition in variable blocks. There were contributions of all categories of the variables: visiting from siblings, visiting from children, talking to whom most in daily life, talking to whom most in daily life, having someone to talk to about what's on your mind, having someone to help in trouble, and having someone to take care of you when sick into the block “social contact”, doing housework, participating in outdoor activities, gardening and raising pets, reading books and newspapers, raising domestic animals, playing cards and mahjong, watching TV and listening to the radio, and participating in social activities into the block “social participation” ; smoke, drink, and exercise were combined into the “lifestyle” block.

## RESULTS

### Descriptive statistics

Most of the respondents were in low-skilled occupations before retirement, living with others, having visits from children frequently, and having good contact with others. However, differences between men and women in education, marital status, social participation, and lifestyle were remarkable; for example, between 39.0% and 42.8% of men in the surveys read books and newspapers, yet only between 8.6% and 18.8% of women did so in comparison (Table S1 in the **Online Supplementary Document**).

### Changes in gender differences in cognitive function

The older men scored better than older women in mean cognitive function in all six waves (**Table 1**). 

**Table 1:** Mean cognitive function between older men and women

	2002	2005	2008	2011	2014	2018
Men	25.56	26.51	25.95	26.50	26.94	27.46
Women	22.59	23.84	23.16	23.74	24.86	25.96
Difference (95% CI)	2.96 (2.78, 3.14)	2.67 (2.48, 2.86)	2.80 (2.60, 3.00)	2.86 (2.52, 3.01)	2.09 (1.74, 2.43)	1.50 (1.27, 1.73)
*P*-value	<0.001	<0.001	<0.001	<0.001	<0.001	<0.001
Relative difference (95% CI)	13.09 (12.30, 13.90)	11.19 (10.40, 12.00)	12.09 (11.23, 12.96)	11.64 (10.64, 12.68)	8.39 (7.00, 9.78)	5.79 (4.89, 6.66)
*P*-value	<0.001	<0.001	<0.001	<0.001	<0.001	<0.001

CI – confidence interval

In reference to 2002, the gender gaps in cognitive function difference between older men and women decreased by 0.29 (95% confidence interval (CI) = -0.49, -0.09) points in 2005, 0.16 (95% CI = -0.43, 0.12) points in 2008, 0.19 (95% CI = -0.60, 0.21) in 2011, 0.87 (95% CI = -1.36, -0.38) in 2014, and 1.46 (95% CI = -1.84, -1.10) in 2018 (**Table 2**and Table S2 in the **Online Supplementary Document**). It is worth noting that, except for the year 2014, the contribution of the time-constant error term to the change in the gap was very low, which indicates that the model was well-designed.

**Table 2:** Percentages of factors contributing to change component: endowments

	Estimate (95% confidence interval)				
	**2005**	**2008**	**2011**	**2014**	**2018**
**Education**	2.74 (-28.16, 33.64)	15.31 (-481.61, 512.23)	1.91 (-44.71, 48.53)	1.66 (-4.78, 8.10)	2.98 (-3.28, 9.23)
**Occupation before retirement**	0.93 (-25.17, 27,03)	-4.28 (-328.06, 319.51)	-4.35 (-25.85, 17.15)	0.37 (-2.26, 3.00)	1.59 (0.02, 3.16)*
**Current residence**	-2.12 (-60.90, 55.66)	4.52 (-413.68, 422.73)	-5.94 (-45.41, 33.53)	1.55 (-8.34, 11.44)	-0.98 (-4.66, 2.69)
**Co-residence**	-0.08 (-2.50, 2.34)	-0.71 (-28.22, 26.81)	0.72 (-12.37, 13.81)	0.46 (-2.21, 3.05)	0.33 (-0.79, 1.45)
**Marital status**	-0.94 (-22.73, 20.85)	-1.16 (-150.70, 148.38)	-8.25 (-55.34, 38.84)	-1.79 (-9.26, 5.67)	-1.34 (-7.78, 5.11)
**Disability**	23.95 (-25.02,7 2.91)	73.99 (27.82, 120.16)	47.22 (-160.76, 255.209	19.45 (5.59, 33.31)†	11.39 (4.89, 17.88)‡
**Life satisfaction**	-0.24 (-6.60, 6.13)	0.01 (-34.06, 34.08)	-0.58 (-8.28, 7.11)	0.36 (-3.97, 4.69)	0.14 (-2.69, 2.97)
**Age (reference: 65-70)**					
71-75	-0.37 (-14.27, 13.53)	0.71 (-29.62, 31.05)	-3.07 (-23.88, 17.75)	-1.47 (-6.25, 3.32)	-0.45 (-1.50, 0.61)
76-80	0.68 (-4.18, 5.55)	0.12 (-6.99, 7.24)	-0.34 (-1.74, 1.60)	0.32	-0.68
81-85	-4.79 (-16.19,6.62)	-9.91 (-26.77, 6.95)	-14.62 (-34.30, 5.06)	-6.53 (-14.78, 1.71)	-4.05 (-7.13, -0.97)‡
86-90	-4.74 (-15.95, 6.46)	-12.99 (-28.25, 2.28)	-19.85 (-45.14, 5.45)	-6.35 (-15.71, 3.01)	-2.06 (-5.48, 1.36)
91-95	-1.25 (-8.51, 6.00)	-22.31 (-61.32, 16.69)	-28.15 (-69.12, 12.82)	0.07 (-15.08, 15.22)	3.99 (-2.28, 10.26)
96-100	1.07 (-1.72, 3.85)	-42.03 (-108.57, 24.50)	-1.14 (-8.44, 6.17)	8.90 (-5.26, 23.07)	10.88 (6.40, 15.37)‡
101-105	68.59 (-9.30, 146.46)	136.63 (-100.84, 374.10)	211.44 (-75.42, 498.30)	79.85 (19.08, 140.63)†	35.87 (26.80, 44.95)‡
**Social participation**	50.96 (-24.25, 126.18)	152.98 (-95.10, 401.07)	93.70 (-32.60, 219.99)	46.68 (20.37, 72.99)‡	31.61 (21.67, 41.56)‡
**Social contract**	-7.92 (-22.85, 7.01)	-12.69 (-55.62, 30.25)	-9.08 (-34.31, 16.15)	-0.27 (-17.51, 16.96)	29.88 (-13.74, 73.51)
**Lifestyle**	7.42 (-19.11, 33.95)	18.62 (-18.48, 55.72)	28.58 (-12,29, 69.45)	5.91 (2.96, 8.85)†	3.88 (1.33, 6.43)†
**Total**	133.90 (72.87, 194.95)	296.82 (136.81, 456.86)†	288.21 (146.94, 431.30)*	149.17 (112.91, 185.37)‡	122.99 (76.08, 169.83)‡

CI – confidence interval

*P-value <0.05.

†*P*-value <0.01.

‡*P*-value <0.001.

In Table 2, the sections labelled "Decomposition" and "Decomposition (%)" illustrate the coefficients and proportions of various components - namely endowment, coefficient, interaction, and random effects. Of particular interest is the endowment effect, as it indicates the extent to which specific explanatory factors contribute to the gaps in cognitive function over time between the two genders. As shown in Table 3, the "Endowment" and "Endowment (%)" rows present the coefficients and proportions of the cognitive function gaps that can be accounted for by variations in individual characteristics. 

**Table 3:** Decomposition of the change of cognitive function gap between older men and women

	Estimate (95% CI)				
	**2005**	**2008**	**2011**	**2014**	**2018**
Change	-0.29 (-0.49, -0.09)*	-0.16 (-0.43, 0.12)	-0.19 (-0.60, 0.21)	-0.87 (-1.36, -0.38)‡	-1.46 (-1.84, -1.10)‡
	**Decomposition (95% CI)**				
Endowments	-0.39 (-0.56, -0.21)‡	-0.47 (-0.72, -0.22)‡	-0.56 (-0.83, -0.28)‡	-1.30 (-1.62, -0.98)‡	-1.79 (-2.47, -1.11)‡
Coefficients	0.12 (-0.15, 0.39)	0.41 (0.09, 0.72)†	0.57 (0.31, 0.83)*	0.94 (0.35, 1.53)†	0.10 (-0.87, 1.06)
Interactions	-0.01(-0.21, 0.22)	-0.08 (-0.27, 0.11)	-0.15 (0.35, 0.05)	-0.40 (-0.85, 0.05)	0.27 (-0.97, 1.50)
Random effects	-0.03 (-0.24, 0.19)	-0.02 (-0.22, 0.19)	-0.06 (-0.32, 0.21)	-0.11 (-0.37, 0.14)	-0.03 (-0.25, 0.19)
	**Decomposition as %, (95% CI)**				
Endowments (%)	133.90 (72.87, 194.95)*	296.82 (136.81, 456.86)†	288.21 (146.94, 431.30)*	149.17 (112.91, 185.37)‡	122.99 (76.08, 169.83)‡
Coefficients (%)	-41.36 (-133.41, 51.06)	-258.55 (-457.95, -58.51)*	-295.60 (-432.46, -160.29)*	-107.83 (-175.77, -39.85)*	-6.69 (-72.81, 59.48)
Interaction (%)	-1.49 (-75.31, 72.54)	50.30 (-71.57, 171.57)	78.82 (-24.83, 182.34)	45.72 (-5.61, 97.00)	-18.32 (-103.01, 66.45)
Random effects (%)	8.95 (-66.28, 84.28)	11.43 (-118.86, 141.65)	28.58 (-108.60, 165.60)	12.95 (-5.61, 97.00)	2.02 (-103.01, 16.81)

CI – confidence interval

*P-value <0.05.

†*P*-value <0.01.

‡*P*-value <0.001.

The coefficients for the endowment effects in 2005, 2008, 2011, 2014, and 2018 were -0.39 (95% CI = -0.49, -0.09), -0.47 (95% CI = -0.72, -0.22), -0.56 (95% CI = -0.83, -0.28), -1.30 (95% CI = 1.62, -0.98), and -1.79 (95% CI = -2.47, -1.11), respectively. The corresponding proportions were 133.90% (95% CI = 72.87, 194.95), 296.82% (95% CI = 136.81, 456.86), 288.21% (95% CI = 146.94, 431.30), 149.17% (95% CI = 112.91, 185.37), and 122.99% (95% CI = 76.08, 169.83). The average of this effect across all years was 178.22%, indicating that the changing endowments between older men and women contribute significantly to explaining the changes in the gaps in their cognitive function. The remaining parts, including coefficients and interactions, were “unexplained”, which may potentially include biological factors.

### Factors attributed to the decomposition of change

**Table 3** presents the detailed breakdown of the contribution proportions of different variables to the explained portion of the change of gender gaps in cognitive function. The results showed that social participation is the most prominent contributor to the variations in gender gaps in cognitive functions (**Table 2**). Specifically, when comparing later years with 2002, we observed that the enhanced levels of social participation among older women have accounted for a significant decrease in cognitive function gaps by 50.96% (95% CI = -24.25, 126.18) for 2005, 152.98% (95% CI = -95.10, 401.07) for 2008, 93.70% (95% CI = -32.60, 219.99) for 2011, 46.68% (95% CI = 20.37, 72.99) for 2014, and 31.61% (95% CI = 21.67, 41.56) for 2018. These contribution proportions were substantial and significant in 2011, 2014, and 2018. Occupation before retirement accounted for 1.59% (95% CI = 0.02, 3.16) of the reduction in gender gaps in cognitive function in 2005, -4.28% (95% CI = -328.06, 319.51) in 2008, -4.35% (95% CI = -25.85, 17.15) in 2011, 0.37% (95% CI = -2.26, 3.00) in 2014, and 1.59% (95% CI = 0.02, 3.16) in 2018. Disability was another significant explanatory variable, accounting for 23.95% (95% CI = -25.02, 72.91) of the reduction in 2005, 73.99% (95% CI = 27.82, 120.16) in 2008, 47.22% (95%CI = -160.76, 255.20) in 2011, 19.45% (95% CI = 5.59, 33.31) in 2014, and 11.39% (95% CI = 4.89, 17.88) in 2018, as did healthy lifestyles, accounting for accounted for 5.91% (95% CI = 2.96, 8.85) in 2014 and 3.89% (95% CI = 1.33, 6.43) in 2018. Occupation before retirement also made a significant contribution to the reduction of gender gaps in cognitive function, accounting for 1.59% (95% CI = 0.02, 3.16) of the difference in 2018. In turn, marital status appeared to have an adverse effect, increasing gender gaps in cognitive function and explaining -0.94% (95% CI = -22.73, 20.85) of the variation in 2005, -1.16% (95% CI = -150.70, 148.38) in 2008, -8.25% (95% CI = -55.34, 38.84) in 2011, -1.79% (95% CI = -9.26, 5.67) in 2014, and -1.34% (95% CI = -7.78, 5.11) in 2018, respectively. However, these effects were not statistically significant, and their significance did not reach the threshold of statistical relevance. Variables were determined to have an ambiguous effect on the reduction of gender differences in cognitive function (i.e. the gap may decrease or amplify depending on the time frame) were not the main focus of our analysis.

### DISCUSSION

We used data from six waves spanning from 2002 to 2018 and employed a longitudinal data decomposition approach to investigate the factors contributing to gender gaps in cognitive function among Chinese older adults. According to our knowledge, this is the first study of its kind conducted in this manner. Findings revealed a cognitive function disadvantage among older women. Moreover, we observed gender gaps in cognitive function exhibited a declining trend over the course of temporal progression.

Currently, the overall level of cognitive function among older women in China is lower than that of men. Previous studies have focused on this issue [1-3], and similar findings of cognitive function disadvantage among women have been observed in many low-income and middle-income countries [34,35]. However, empirical studies conducted in developed countries mostly indicated that older women perform similarly or better than men in cognitive tests [36,37]. Weber et al. [25] demonstrated that in less advantaged areas (characterized by relatively lower gross domestic product, higher mortality rates, larger family size, and lower educational levels), both men and women displayed poorer cognitive function, with the latter group showing lower levels. These findings may partly explain the contrasting patterns of gender gaps in cognitive function among older adults in different countries.

Encouragingly, we observed a trend of decreasing gender gaps in cognitive function. From 2002 to 2018, the gender gaps in cognitive function among older men and women decreased by 1.455 points. A recent study using data from multiple countries (USA, Europe (including UK and Ireland), South Korea, China, Mexico, and Costa Rica) found that women showed a more significant improvement in cognitive function over time compared to men [38]. In China, differences in gender roles, opportunities, and obligations determine the experiential differences between men and women during their lifetime. As Chinese society progresses, the macro-social background, along with gender norms and welfare systems, creates opportunities for cognitive reserves to accumulate during early (e.g. through education) and late life (eg, through gendered expectations of caregiving and women's labor market participation) [25,39]. China has undergone significant transformations across different periods, leading to improvements in women's living conditions and socioeconomic status. For instance, opportunities for women's education [40] and gender ideologies [41] as driving factors of gender gaps in cognitive function [42] have greatly contributed to the improvement of their cognitive function in the context of historical changes in China. As China's younger population ages in the future, the gender gap in cognitive function among older women may further diminish, owing to the enhancements in their living conditions and socioeconomic status.

Lifestyle was also found as a significant contributing factor to narrowing the gap in cognitive functioning in this study. Over time, older women have been able to maintain increasingly healthier lifestyles over time compared to older men. Previous studies have also shown that women were more likely to adhere to healthy lifestyle behaviors in China [43,44]. This adherence may be attributed to socio-cultural factors. For instance, traditional Chinese culture encourages smoking and drinking among men, and men who engage in these behaviors are considered masculine. However, Chinese culture does not encourage women to do the same [45].

Education, occupational achievement, and social participation are considered three important factors closely related to cognitive function levels in the cognitive reserve theory [46]. We found that, compared to education and occupation, social participation made a more significant contribution to explaining the changes in cognitive function gaps between older Chinese men and women. Possible mechanisms of the positive influence of social participation on cognitive function include the association between social activities and improved psychological well-being [47], the promotion of healthy lifestyles through social participation [48], the expansion of social networks, and the maintenance of cognitive reserve [49,50], ultimately leading to better cognitive functioning in later life. Previous research has also indicated that gender differences in social participation are gradually diminishing in China [51], suggesting that women experience greater improvements in cognitive functioning through social participation within the context of social development, ultimately contributing to the narrowing of gender gaps.

Our findings signal several implications for addressing the observed lower level of cognitive function among older women compared to men. First, it is crucial for families, governmental agencies, and society as a whole to create more opportunities for elder women to engage in social activities. This could include encouraging regular family visits and establishing more senior activity centers within their communities. Second, it is vital to implement interventions that promote healthier behaviors, given their importance in addressing gender gaps. Such interventions could encompass health education programs designed to enhance knowledge and raise awareness about healthy lifestyle choices. Lastly, as older women often experience poorer physical health than older men – a factor that may contribute significantly to the gender disparity in cognitive function, it is critical to strengthen rehabilitation services for disabled older women. Additionally, enhancing health management services specifically tailored for older women is equally essential.

Some limitations need to be cautiously considered when interpreting our findings. Firstly, our study relies on self-reported data, which may be subject to reporting bias. However, studies have found the data quality of the CLHLS to be adequate [52,53]. Secondly, this research is based this research on tracking data; like any longitudinal study, there is a possibility of potential bias due to data attrition. However, CLHLS renews the survey sample in each wave to ensure balance. Furthermore, gender gaps in cognition function are the result of the complex interaction of biological, psychological, and social factors across life course. Unmeasured confounding factors could have influenced our analysis, since we were unable to consider health behaviors during early life stages, including childhood and adulthood, and other unmeasured confounders. At last, the change in cognitive performance between older men and women should be understood because of complex contextual and structural phenomena; thus, future research should adopt an interdisciplinary design to explore the underlying mechanisms between the various factors and cognitive function, and also conduct more detailed subgroup analyses.

## CONCLUSIONS

We employed the extension of the KOB decomposition approach to longitudinal data to decompose gender gaps and investigate the changes in cognitive function between older men and women in China. Our findings consistently demonstrated that older women had lower cognitive function compared to older men across all periods. However, we observed a narrowing trend in this gap. Notably, among the factors influencing the reduction, social participation emerged as a prominent modifiable social determinant. Our findings underline the importance of prioritizing interventions aimed at promoting social participation among older adults to enhance their cognitive function.

## Additional material


Online Supplementary Document
