# Supplementary material for: Decomposing the change in the cognitive function gap between older men and women over time in China: The Chinese Longitudinal Healthy Longevity Survey
Source: J Glob Health. 2023 Nov 22;13:04143. doi: 10.7189/jogh.13.04143 (PMC10662781; doi:10.7189/jogh.13.04143)
Supplement: Online Supplementary Document [file jogh-13-04143-s001.pdf]

ONLINE SUPPLEMENTARY DOCUMENT

Title: Decomposing the Change in the Cognitive Function Gap Between Older Men and Women Over Time in China: The Chinese Longitudinal Healthy Longevity Survey

Authors: Na Cao[1], Xin Zeng[1], Peigang Wang[1*]

Affiliations: [1] Department of Social Medicine and Management, School of Public Health, Wuhan University, Wuhan, Hubei, China.

Contents

Appendix S1. Measurement of the independent variable 3

Appendix S2. Method 5

Table S1. Descriptive statistics by gender and period 7

Table S2. Decomposition of cognitive function gap between elder men and women 9

Appendix S1. Measurement of the independent variable

Based on previous research, the independent variables included age, education, occupation before retirement, marital status, current residence, co-residence, disability, social participation(doing housework, participating in outdoor activities, gardening and raising pets, reading books and newspapers, raising domestic animals, playing cards or mahjong, watching TV and listening to the radio, and participating in social activities), social contact (visiting from siblings, visiting from children, talking to someone, having someone to talk to about what's on your mind, having someone to help in trouble, having someone to take care of you when sick), lifestyle (smoking, drinking, exercising), and life satisfaction.

We divided age into eight age groups: 65-70，71-75，76-80，81-85，86-90，91-95，96-100， and 101-105. Education was coded as illiterate (had not received any education) and literate. Primary occupation before retirement was coded as low-skilled occupation and high-skilled occupation. Low-skilled occupations included agriculture, worklessness, domestic work, freelancing and others. High-skilled occupations included professionals, doctors, teachers, administrators, general clerks, service workers, labourers, and military personnel. Marital status was coded as married and not married. The current residence was dichotomized as urban and rural. The co-residence was coded as living alone and living with others. Disability was measured by the Activities of Daily Living scale, but any item that was not completed is considered disabling, otherwise, it was not. Lifestyles included smoking, drinking, and exercising. The three variables were measured by "Do you do you smoke at present?" "Do you drink at present?" "Do you exercise regularly at present?". Options are coded as "yes" or "no" for all three variables.

Social participation included the following eight items: doing housework (cooking, taking care of kids, etc.), participating in outdoor activities(personal), gardening and/or raising pets, reading books and/or newspapers, raising domestic animals, playing cards and/or mahjong, watching TV and/or listening to the radio, and participating in social activities(organized). Options included almost every day, not every day but at least once a week, not every week but at least once a month, and not every month but sometimes. The options were combined as never attend and attend.

Social contact was measured by six questions: "Do your siblings visit you frequently?", "Do your children visit you frequently? , "To whom do you usually talk most frequently in daily life?" , " To whom do you talk first when you need to tell something of your thoughts?" , " Who do you ask first for help when you have problems/difficulties?" and " When you are sick, who usually takes care of you?". The options for the first two questions were: Not frequently and frequently. The options for the last four questions were combined as " nobody to talk to" and "someone to talk to"; " nobody to talk to" and "someone to talk to"; " nobody to solve" and "someone to solve"; and " nobody to take care of" and "someone to take care of".

Life satisfaction was measured by the item “How do you rate your life at present?” Options included 1 (very good), 2 (better), 3 (fair), 4 (worse), and 5 (very bad). According to the distribution of the data, those who chose "very good", "relatively good" and "average" were combined into "good" and those who answered the other two were combined into "not good".

Appendix S2. Method

Since panel regressions model time-constant individual error terms, a decomposition using panel regression models must take empirical group differences in this time-constant, unobserved variables into account. Thereby, the time constant individual error terms μ^l become part of the decomposition of group-level differences. We start with a basic mixed linear regression model for an outcome Y and two groups A and B:

$$Y_t^l = X_t^l \beta_t^l + \mu^l + \epsilon_t^l, \qquad E(\epsilon_t^l) = 0, COV(X_t, \epsilon_t) = 0 \qquad l \in [A, B]$$

The outcome difference can be decomposed into following 4 components.

$$\Delta Y_t = E_t + C_t + I_t + U$$

$$E_t = \{E(X_t^A) - E(X_t^B)\}\beta_t^B$$

$$C_t = E(X_t^B)(\beta_t^A - \beta_t^B)$$

$$I_t = \{E(X_t^A) - E(X_t^B)\}(\beta_t^A - \beta_t^B)$$

$$U = \{E(u^A) - E(u^B)\}$$

The first component (E) amounts to the part of the difference that is due to group differences in the predictors (so-called endowments effect). The second component (C) measures the contribution of differences in the coefficients. And the third component (I) is an interaction term accounting for the fact that differences in endowments and coefficients exist simultaneously between the two groups. Taking the time-constant error terms into account adds the differences in the expectation of μ^las a fourth component U to the decomposition. This component is not time-dependent. It only comprises differences between groups in the time-constant error terms. In a well-designed model, the proportion of random effects should be low (or more precisely, close to zero) [1].

In this study, we take the form of a threefold decomposition- the interventionist approach(Kröger and Hartmann argue that the interventionist approach is best suited to address a certain kind of research question which regularly arises in applied social science research and similar fields like epidemiology or public health) [2]. The change in the outcome difference between the two groups is given by

$$\Delta Y^A - \Delta Y^B = \Delta Y = \Delta E + \Delta I + \Delta C$$

To obtain the endowments component, coefficients component, and the interactions between the change in endowments and coefficients, we allow only the groups' composition to vary over time and hold the coefficients constant at their initial group-specific levels at times. The calculations for the changes in each component between the two groups and between the two points in time are shown in the equations below.

$$\begin{aligned}\Delta E &= E(X_t^A)\beta_s^A - E(X_s^A)\beta_s^A - E(X_t^B)\beta_s^B + E(X_s^B)\beta_s^B \\ &= [E(X_t^A) - E(X_s^A)]\beta_s^A - [E(X_t^B) - E(X_s^B)]\beta_s^B \\ \Delta C &= E(X_s^A)\beta_t^A - E(X_s^A)\beta_s^A - E(X_s^B)\beta_t^B + E(X_s^B)\beta_s^B \\ &= E(X_t^A)(\beta_t^A - \beta_s^A) - E(X_s^B)(\beta_t^B - \beta_s^B)\end{aligned}$$

$$\Delta I = [E(X_t^A) - E(X_s^A)](\beta_t^A - \beta_s^A) - [E(X_t^B) - E(X_s^B)](\beta_t^B - \beta_s^B)$$

References

1 Zdeněk R, Lososová J, Svoboda J. Are the incomes of agricultural households lagging behind? Evidence from Czechia. Economic Research-Ekonomska Istraživanja. 2022;35:7066–83.

2 Kröger H, Hartmann J. Extending the Kitagawa–Oaxaca–Blinder decomposition approach to panel data. The Stata Journal. 2021;21:360–410.

Table S1. Descriptive statistics by gender and period

(Proportion)

Variables	Category	2002		2005		2008		2011		2014		2018	
		Me	Wo	Me	Wo	Me	Wo	Me	Wo	Me	Wo	Me	Wo
**Education**	Illiterate	33	80	32	78	34	79	30	75	28	70	21	55
	Literate	66	19	67	21	65	20	69	24	72	29	78	44
**Occupation before retirement**	Low-	66	86	67	85	71	87	72	87	72	85	70	81
	High-	33	13	32	14	28	12	27	12	27	14	29	18
**Current residence**	Rural	53	54	54	57	59	59	52	50	41	39	40	43
	Urban	46	45	45	43	40	40	47	49	58	60	59	56
**Co-residence**	Live	11	15	12	15	15	18	14	19	15	21	14	21
	Live with	88	84	87	84	84	81	85	80	84	78	85	79
**Marital status**	Not	47	79	45	76	46	77	40	73	39	69	33	59
	Married	52	21	54	23	53	23	59	27	61	30	66	40
**Social contact**													
**Visit from siblings**	Not	73	80	65	74	52	63	44	54	54	60	49	54
	Frequentl	26	19	34	25	47	36	55	45	46	39	50	45
**Visit from children**	Not	14	15	4.	3.	11	13	10	10	7.	5.	1.	2.
	Frequentl	86	84	95	96	88	86	90	89	92	94	99	97
**Talk to whom most in daily life**	No	4.	5.	3.	3.	3.	4.	3.	4.	3.	3.	2.	2.
	Yes	95	94	97	96	96	95	96	95	96	96	98	97
**Have someone to talk to about what's on your**	No	6.	6.	3.	3.	3.	4.	5.	5.	4.	4.	2.	3.
	Yes	93	93	96	96	96	96	94	94	95	95	97	96
**Have someone to help in trouble**	No	3.	2.	1.	2.	1.	1.	3.	2.	2.	2.	1.	1.
	Yes	96	97	98	98	98	98	97	97	97	97	98	98
**Have someone to take care of you when sick**	No	2.	2.	1.	2.	2.	2.	2.	2.	1.	2.	2.	2.
	Yes	97	97	98	97	97	97	97	98	98	97	97	97
**Social participation**													
**Housework**	No	43	39	39	34	40	35	40	34	39	28	34	24
	Yes	56	60	60	66	59	64	59	65	60	71	65	75
**Outdoor activities**	No	21	34	22	32	26	35	25	35	28	34	2.	3.
	Yes	78	65	77	67	73	64	74	64	71	65	98	97
**Raise domestic animals**	No	78	86	77	85	82	87	73	79	73	75	73	76
	Yes	21	13	22	14	17	12	26	20	26	24	26	23
**Read books and newspaper**	No	59	91	59	89	67	91	61	89	58	85	57	81
	Yes	40	8.	40	10	32	8.	39	10	41	14	42	18
**Garden and raise pets**	No	71	71	69	69	72	72	70	75	73	74	72	71
	Yes	28	28	30	30	27	27	29	24	26	26	27	28
**Play cards and mahjong**	No	74	86	73	85	77	86	76	86	73	84	73	80
	Yes	26	13	26	15	22	13	23	13	26	15	26	20
**Watch TV and listen to the radio**	No	22	38	17	31	18	31	15	30	12	24	11	21
	Yes	77	61	82	68	81	68	84	69	87	75	88	79
**Social activities**	No	78	89	77	87	82	89	78	86	76	83	76	81
	Yes	21	10	22	12	17	10	21	13	24	16	23	18
**Lifestyle**													
**Smoke**	No	65	92	62	92	65	93	64	94	66	94	68	95
	Yes	34	7.	37	7.	34	6.	35	5.	33	6.	31	4.
**Drink**	No	66	88	66	88	69	92	70	91	71	93	71	93
	Yes	33	11	34	11	30	7.	29	8.	28	6.	28	6.
**Exercise**	No	55	72	58	71	61	74	55	64	56	66	53	61
	Yes	44	27	41	28	38	25	44	35	43	34	46	38

**Disability**	No	83	71	88	80	90	84	85	77	84	80	91	88
	Yes	17	28	11	19	9.	15	14	22	15	19	8.	12
**Life satisfaction**	Not good	38	37	39	37	40	40	38	36	31	32	29	28
	Good	61	62	60	62	59	59	61	63	68	67	70	71
**Age group**													
**65-70**		15	11	16	13	14	10	10	7.	1.	0.	19	18
**71-75**		13	10	14	11	12	10	17	14	19	20	17	15
**76-80**		12	10	13	10	12	10	16	13	25	21	16	16
**81-85**		17	12	11	9.	15	12	16	14	16	16	15	15
**86-90**		14	12	18	15	17	15	14	13	17	16	11	11
**91-95**		13	13	14	14	15	16	14	15	11	11	11	10
**96-100**		6.	10	7.	10	6.	11	6.	10	7.	9.	4.	5.
**101-105**		6.	18	4.	13	4.	13	3.	9.	1.	3.	3.	7.
**N**		58	72	56	66	57	67	29	33	16	16	32	35

Table S2. Decomposition of cognitive function gap between elder men and women

	2002	2005	2008	2011	2014	2018
	Estimate [95% CI]	Estimate [95% CI]	Estimate [95% CI]	Estimate [95% CI]	Estimate [95% CI]	Estimate [95% CI]
**Observed**	2.96***	2.67***	2.80***	2.76***	2.09***	1.50***
	[2.78,3.14]	[2.48,2.86]	[2.60,3.00]	[2.52,3.01]	[1.74,2.43]	[1.27,1.73]
**Decomposition**						
**Endowments**	2.33***	2.11***	1.93***	2.04***	1.31***	1.02***
	[2.07,2.60]	[1.80,2.41]	[1.61,2.25]	[1.75,2.33]	[0.89,1.73]	[0.83,1.20]
**Coefficients**	0.48*	0.79***	0.91***	1.04***	1.003***	0.56**
	[0.10,0.86]	[0.58,1.00]	[0.67,1.16]	[0.66,1.43]	[0.55,1.46]	[0.22,0.89]
**Interactions**	0.13	-0.22	-0.04	-0.28	-0.13	-0.06
	[-0.22,0.48]	[-0.50,0.07]	[-0.31,0.23]	[-0.68,-0.13]	[-0.49,0.24]	[-0.29,0.18]
**Random effects**	0.02	-0.01	-0.00	-0.04	-0.10	-0.01
	[-0.12,0.15]	[-0.09,0.07]	[-0.12,0.12]	[-0.28,0.20]	[-0.29,0.10]	[-0.11,0.08]
**Total**	2.96***	2.67***	2.80***	2.76***	2.09***	1.50***
	[2.78,3.14]	[2.48,2.86]	[2.60,3.00]	[2.52,3.01]	[1.74,2.43]	[1.27,1.73]
**Decomposition (%)**						
**Endowments (%)**	78.85***	78.89***	68.83***	73.74***	62.66***	67.63***
	[70.51,87.18]	[69.56,88.23]	[58.39,79.27]	[67.12,80.37]	[44.82,80.51]	[48.92,86.34]
**Coefficients (%)**	16.19**	29.54***	32.61***	37.68***	48.08***	37.05***
	[3.94,28.44]	[22.8,36.28]	[25.51,39.7]	[24.20,51.16]	[25.42,70.75]	[17.73,56.36]
**Interactions (%)**	4.43	-8.06	-1.36	-9.99	-6.10	-3.78
	[-7.27,16.13]	[-18.33,2.21]	[-11.11,8.39]	[-23.97,3.99]	[-23.80,11.59]	[-20.96,13.41]
**Random effects (%)**	0.54	-0.38	-0.08	-1.43	-4.65	-0.90
	[-3.88,4.95]	[-3.34,2.59]	[-4.52,4.37]	[-10.02,7.17]	[-15.20,5.91]	[-7.18,5.38]
